# Histamine Ingestion by *Anopheles stephensi* Alters Important Vector Transmission Behaviors and Infection Success with Diverse *Plasmodium* Species

**DOI:** 10.3390/biom11050719

**Published:** 2021-05-11

**Authors:** Anna M. Rodriguez, Malayna G. Hambly, Sandeep Jandu, Raquel Simão-Gurge, Casey Lowder, Edwin E. Lewis, Jeffrey A. Riffell, Shirley Luckhart

**Affiliations:** 1 Department of Entomology, Plant Pathology and Nematology, University of Idaho, Moscow, ID 83843-3051, USA; amrodriguez@uidaho.edu (A.M.R.); hamb8545@vandals.uidaho.edu (M.G.H.); rsimaogurge@uidaho.edu (R.S.-G.); lowder@uidaho.edu (C.L.); eelewis@uidaho.edu (E.E.L.); 2 Department of Biology, University of Washington, Seattle, WA 98195-1800, USA; sjandu1@uw.edu (S.J.); jriffell@uw.edu (J.A.R.); 3 Department of Biological Sciences, University of Idaho, Moscow, ID 83843-3051, USA

**Keywords:** histamine, *Anopheles stephensi*, flight behavior, blood-feeding behavior, infection success, lifespan, *Plasmodium yoelii*, *Plasmodium falciparum*

## Abstract

An estimated 229 million people worldwide were impacted by malaria in 2019. The vectors of malaria parasites (*Plasmodium* spp.) are *Anopheles* mosquitoes, making their behavior, infection success, and ultimately transmission of great importance. Individuals with severe malaria can exhibit significantly increased blood concentrations of histamine, an allergic mediator in humans and an important insect neuromodulator, potentially delivered to mosquitoes during blood-feeding. To determine whether ingested histamine could alter *Anopheles stephensi* biology, we provisioned histamine at normal blood levels and at levels consistent with severe malaria and monitored blood-feeding behavior, flight activity, antennal and retinal responses to host stimuli and lifespan of adult female *Anopheles stephensi*. To determine the effects of ingested histamine on parasite infection success, we quantified midgut oocysts and salivary gland sporozoites in mosquitoes infected with *Plasmodium yoelii* and *Plasmodium falciparum*. Our data show that provisioning *An. stephensi* with histamine at levels consistent with severe malaria can enhance mosquito behaviors and parasite infection success in a manner that would be expected to amplify parasite transmission to and from human hosts. Such knowledge could be used to connect clinical interventions by reducing elevated histamine to mitigate human disease pathology with the delivery of novel lures for improved malaria control.

## 1. Introduction

In 2019, there were an estimated 229 million cases of malaria worldwide [1]. *Anopheles* mosquitoes are the vectors of malaria parasites (*Plasmodium* spp.), making their behavior, infection success, and ultimately their role in parasite transmission of great importance. Both children and adults with severe falciparum malaria (SFM) can exhibit significantly increased levels of blood histamine [2,3], which is generated from the amino acid histidine by the catalytic action of histidine decarboxylase (HDC), which is expressed primarily by basophils and mast cells. Strikingly, blood histamine in pediatric SFM can rise to nearly 10 nM or up to 480% of uninfected levels [2,3]. Increased levels of this allergic mediator in blood are notable in that histamine is a significant contributor to inflammation and disease pathology not only in malaria [2,3], but also in many other disease states [4].

In addition to its role in malaria pathology, histamine is an important insect neuromodulator, highlighting the potential that this biogenic amine is ingested at bioactive levels by mosquitoes feeding on gametocytemic individuals. In anopheline mosquitoes, transcripts for histamine receptors and HDC are expressed in the midgut and head [5,6]. Across insect species, strong abdominal histamine receptor expression and histaminergic neurons project to the brain, where they innervate sensory brain regions to influence behavior [7,8,9]. In a wide variety of arthropods, signaling between the gut-brain and abdomen-brain has been shown to facilitate the processing of food and biologically important sensory (visual and olfactory) information [7,8,9]. Histaminergic signaling between thoracic and brain regions has also been shown to be involved in sensory modulation and flight [8,10,11]. The anopheline mosquito represents a unique situation to explore gut-brain axis biology in that these mosquitoes would be predicted to ingest varying levels of histamine at blood-feeding, bringing these compounds into direct contact with mosquito tissues, including the midgut, that express receptors and biosynthetic enzymes for biogenic amines.

While it is true that increased gametocytogenesis has been correlated with SFM, specifically with patterns of increased host immunity, severe anemia, and induced reticulocytosis [12,13,14,15,16], the presence of gametocytes does not always predict successful transmission to mosquito vectors. Further, while malarial gametocytemia has been positively correlated with mosquito infectivity [17,18,19], gametocyte commitment can occur without the maturity to infectious stage V gametocytes, and gametocytemia is not the only predictor of mosquito infectivity [20,21,22,23]. In this context, we sought to test the hypothesis that malaria-associated changes to circulating histamine in host blood and its effects on mosquito biology could facilitate successful infection of anopheline mosquitoes by gametocytemic blood.

In these studies, we provisioned histamine at normal blood levels (1 nM) and levels consistent with severe malaria (10 nM) and monitored blood-feeding behavior, flight activity, antennal, and retinal responses to host stimuli, and lifespan of adult female *Anopheles stephensi*, a globally important and highly invasive malaria mosquito vector that has become established in Africa [24,25,26,27]. We also evaluated the impacts of ingested histamine on *An. stephensi* infection with the mouse parasite *Plasmodium yoelii yoelii* 17XNL and the human parasite *Plasmodium falciparum*. Our data show that provisioning *An. stephensi* with histamine at levels consistent with severe malaria can enhance parasite infection and mosquito behaviors that would be expected to amplify parasite transmission to and from human hosts. Such knowledge could be used to connect clinical interventions (e.g., reducing elevated histamine to mitigate human disease pathology [28]) with the delivery of novel lures to manipulate histamine signaling in vector mosquitoes for improved future malaria control.

## 2. Materials and Methods

### 2.1. Materials

Histamine (2-(1H-imidazol-5-yl)ethanamine; dihydrochloride, ACROS Organics, Fair Lawn, NJ, USA), human red blood cells (RBCs, Interstate Blood Bank, Memphis, TN, USA), and serum (Interstate Blood Bank, Memphis, TN, USA), RPMI-1640 medium (Gibco, Gaithersburg, MD, USA) supplemented with HEPES, L-glutamine, hypoxanthine (Acros Organics), DL-lactic acid (Acros Organics), Hoechst 33342-trihydrochloride trihydrate (Invitrogen, Carlsbad, CA, USA), JC-1 (Invitrogen, Carlsbad, CA, USA), mercurochrome, Ringer’s solution (3 mM CaCl_2_, 182 mM KCl, 46 mM NaCl, 10 mM Tris pH 7.2), 1-octen-3-ol (Sigma-Aldrich), and electrode gel (Parker Laboratories, Fairfield, NJ, USA). N-3-dimethylaminopropyl-N′-ethylcarbodiimide (Sigma-Aldrich, St. Louis, MO, USA, cat. #03449), paraformaldehyde (Electron Microscopy Sciences, Hatfield, PA, USA, cat. #15710), agarose (Sigma-Aldrich), goat serum (BSA; Jackson ImmunoResearch Laboratories, West Grove, PA, USA, cat. #001-000-162), rabbit anti-histamine (ImmunoStar, Hudson, WI, USA, cat. # 22939, RRID:AB_572245), Alexa Fluor 488 (Thermo Fisher, cat. #A-11008), and Vectashield^®^PLUS (Vector Laboratories, Burlingame, CA, cat. #H-1900).

### 2.2. Mosquito Rearing

*Anopheles stephensi* Liston, an Indian wild-type strain, were derived from the colony maintained in the Luckhart lab since 1998, at which time they were acquired from the long-term colony maintained in the Department of Entomology at Walter Reed Army Institute of Research (WRAIR, Washington, DC). For these studies, mosquitoes were reared and maintained under a 12 h light-dark cycle and at 27 °C and 80% humidity. Unless otherwise stated, adult mosquitoes were maintained on 10% sucrose-soaked cotton balls in a 1 ft^3^ wire mesh cage. For egg production, the adult females were allowed to feed for 30 min on live mice sedated with ketamine (50 mg/kg) and xylazine (5 mg/kg) in sterile saline. Prior to mosquito feeding, the mice were maintained in standard cages and offered free access to food and water. After the mosquito feeding was complete, the mice were euthanized by CO_2_ inhalation followed by cervical dislocation. All animal procedures were conducted as approved by the Institutional Animal Care and Use Committee of the University of Idaho (protocol IACUC-2020-10, approved 30 March 2020). Mosquitoes were provided oviposition substrates 2 d following the bloodmeal. Collected eggs were washed into 5 L Nalgene pans with shallow water and maintained as larvae in the same pans, on a solution of 2% powdered fish food (Sera Micron) and baker’s yeast in a 2:1 ratio for 3 d and supplemented with Game Fish Chow pellet food (Purina) until pupation. Experimental mosquitoes were 4–6 d old at the beginning of all experiments, collected at least 24 h prior to the beginning of an experiment and housed in a 500 mL polypropylene Nalgene container with mesh screening.

### 2.3. Techniques

#### 2.3.1. Histamine Priming of Female *An. stephensi*

For some studies, histamine was provisioned to mosquitoes through priming as follows. Control and treatment groups consisted of 90–120 female mosquitoes each per two-liter container provided with soaked cotton balls and a sugar cube. Controls were primed with water (control), while treatments were primed with histamine at 1 nM (healthy blood levels) or 10 nM (severe malaria [2,29]) in water via soaked cotton balls. The histamine solutions were made fresh daily, and the soaked cotton balls were changed twice daily, once in the morning between 0830–1000 and once in the evening between 1630–1800. Mosquitoes were primed for 3 d prior to the first bloodmeal. The soaked cotton balls and sugar were removed from the cartons 30 min to 1 h prior to blood-feeding.

#### 2.3.2. Artificial Bloodmeal Delivery to Female *An. stephensi*

Mosquitoes were offered a bloodmeal (washed human RBCs and heat-inactivated serum 1:1 vol:vol) via an artificial feeder and were exposed to the bloodmeal for 15 min. Bloodmeals were offered in the morning between 0700–1100, following lights on at 0700. We maintained this timing for consistency throughout our studies, despite the fact that time of day for blood-feeding has been shown to minimally affect reproduction, without effects on fitness or malaria parasite infection in *An. stephensi* [30]. Between bloodmeals, female mosquitoes were maintained on 10% sucrose-soaked cotton balls.

#### 2.3.3. Histamine Provisioning in a Bloodmeal to Female *An. stephensi*

For some studies, histamine was provisioned to mosquitoes in an artificial bloodmeal as described in Section 2.3.2. Histamine was dissolved in water and diluted into the bloodmeal to final concentrations of 1 nM or 10 nM. An identical control meal was prepared by adding a volume of water (3 μL) equivalent to that used for histamine treatments to 3 mL of the RBC-serum meal.

#### 2.3.4. Mouse Infection with *Plasmodium yoelii yoelii* 17XNL

Female 8–10-week-old CD1 mice were purchased from Envigo (Indianapolis, IN, USA) for parasite infection. Patterns of parasitemia and gametocytemia are indistinguishable between male and female mice, but female mice are larger and easier to manipulate, so female mice were used for these studies. Mice were maintained in standard cages and offered free access to food and water. All animal procedures were conducted as approved by the Institutional Animal Care and Use Committee of the University of Idaho (protocol IACUC-2020-10, approved 30 March 2020). Mice were acclimated for 1 week prior to the beginning of the study. To infect mice, 1 × 10^7^ *P. y. yoelii*-infected RBCs were injected intraperitoneally into each mouse. Mice were monitored daily for parasitemia beginning at 2 d post-infection (PI) via microscopy of thin blood smears stained with Giemsa. Wet prep slides made from a drop of blood were evaluated for exflagellation of male gametocytes and counted as events per field of view prior to mosquito feeding. Mice with similar levels of exflagellation were chosen to infect mosquitoes and were anesthetized with ketamine (50 mg/kg) and xylazine (5 mg/kg) in sterile saline and placed on mosquito cartons for 15 min to allow mosquitoes to feed. After mosquito feeding was complete, mice were euthanized by CO_2_ inhalation followed by cervical dislocation per our approved IACUC protocol.

#### 2.3.5. *P. falciparum* NF54 Culture

*Plasmodium falciparum* NF54 culture was initiated at 0.5%–0.7% parasitemia. Parasites were maintained in RPMI 1640 medium supplemented with 25 mM HEPES, L-glutamine, 49 mM hypoxanthine, and 8.2 mM DL-lactic acid, with 10% heat-inactivated human serum. Parasites were synchronized by treatment with 5% sorbitol as previously described [31,32,33].

### 2.4. Impact of Histamine Provisioning on Uninfected Female An. stephensi Behavior

#### 2.4.1. Tendency to Take a Second Bloodmeal

Mosquitoes were treated with histamine by priming followed by a first bloodmeal (Section 2.3.1) or by delivery of histamine in a first bloodmeal (Section 2.3.3). After blood-feeding, unfed mosquitoes were removed and discarded. The remaining mosquitoes were provided with an oviposition substrate at 3 d post-feeding, which was removed the following day. Mosquitoes were offered a second bloodmeal at 4 d or 14 d after the first. At both 4 d and 14 d, blood-fed and non-blood-fed mosquitoes were recorded. We performed five biological replicates with both histamine priming and histamine delivery in the first bloodmeal, at each time point using separate cohorts of *An. stephensi*.

#### 2.4.2. Flight Activity and Visual Object Investigation in Response to CO_2_

All behavioral experiments were conducted in a low-speed wind tunnel (ELD Inc., Lake City, MN, USA), with a working section 224 cm long × 61 cm wide × 61 cm high provided with a constant laminar flow of 40 cm/s. We used three short-throw projectors (LG PH450U, Englewood Cliffs, NJ, USA) and rear projection screens (SpyeDark, Spye, LLC, Minneapolis, MN, USA) to provide a low contrast checkerboard on the floor of the tunnel and grey horizons on each side of the tunnel. The intensity of ambient light from the projectors was 5 lux across the 420–670 nm range. A 3D real-time tracking system [34,35] was used to track mosquito trajectories. Sixteen cameras (Basler AC640gm, Exton, PA, USA) were mounted on top of the wind tunnel and recorded mosquito trajectories at 60 frames/s. All cameras had an opaque Infrared (IR) Optical Wratten Filter (Kodak 89B, Kodak, Rochester, NY, USA) to mitigate the effect of light in the tracking. IR backlights (HK-F3528IR30-X, LedLightsWorld, Bellevue, WA, USA) were installed below and the sides of the wind tunnel to provide constant illumination beyond the visual sensitivity of the mosquitoes. The temperature within the wind tunnel, measured using ibuttons and FLIR cameras, was a constant 22.5 °C and did not show any variability within the working section [34,36]. Ambient CO_2_ was constantly measured outside of the tunnel and was approximately 410 ppm.

For each assay, 50 female *An. stephensi* were transferred into the tunnel and, after 1 h of acclimation, a 5% CO_2_ plume (or filtered air in control experiments) was released from a point source at the immediate upwind section of the tunnel at a height of 30 cm in the centerline of the tunnel. The CO_2_ remained on for 1 h, after which filtered air was returned to the tunnel for 1 h (post-CO_2_). The CO_2_ and filtered air were delivered using two mass flow controllers (MC-200SCCM-D, Alicat Scientific, Tucson, AZ, USA) controlled by a Python script that allowed synchronizing odor and filtered air delivery with the trajectory behaviors. To examine mosquito responses to visual stimuli, we placed 5 cm diameter white and black paper circles (Color-aid Corp., Hudson Falls, NY, USA) 18 cm apart on the floor of the wind tunnel in a row perpendicular to the direction of airflow.

Our tracking system cannot maintain mosquito identities for extended periods of time, but individual trajectories were assumed to be independent for statistical analysis. Analyses were restricted to trajectories that were at least 90 frames (1.5 s) long. Trajectories that were at least 1.5 s were analyzed (average 3.1 s, longest 28.7 s, *n* = 10,635 trajectories). Flight velocities for each mosquito path were analyzed based on their 3D trajectory for the pre-CO_2_ (filtered air), CO_2_, and post-CO_2_ (filtered air) conditions. To examine mosquito behaviors and preferences to black and white circles in the tunnel, a fictive volume for mosquito primary activity was created around these cues (area 14 × 14 cm, height 4 cm) that was centered over the object in the crosswind direction and shifted slightly downwind in the wind line direction. The percentage of time mosquitoes investigated the objects relative to their time spent flying in the wind tunnel was also determined. In an experimental trial, approximately 10%–25% of the trajectories approached the objects. Experiments were performed with histamine-primed mosquitoes (Section 2.3.1) and mosquitoes provisioned with histamine in an RBC-serum meal (Section 2.3.3).

#### 2.4.3. Electroretinogram (ERG) and Electroantennogram (EAG) Recordings of *An. stephensi* Response to Host-Associated Visual and Odor Stimuli

Electroretinogram (ERG) recordings were performed by fixing individual female *An. stephensi* to a coverslip using Bondic glue. Mosquitoes were dark-adapted prior to stimulation. The recording glass electrode (thin-wall glass capillaries; OD, 1.0 mm; length, 76 mm; World Precision Instruments, Sarasota, FL, USA, cat. # TW100F-3) was pulled using a micropipette puller (Sutter Instrument, Novato, CA, USA, p-2000) and filled with Ringer’s solution. The reference electrode, a sharpened tungsten wire, was placed into one compound eye in a small drop of electrode gel, and the recording electrode was placed immediately on the surface of the contralateral eye. Mosquitoes were placed at the center of a semi-cylindrical visual arena (frosted mylar, 10 cm diameter, 10 cm high); a video projector (Acer K132 WXGA DLP LED Projector) was positioned in front of the arena to project the visual stimuli. To test the response to different colors, we used 2 s pulses of red, blue, or green light (distinct peaks at 460, 535, and 630 nm, 70 μW/cm^2^/nm), with 30 s of no light (black) between each stimulus. The colors were presented in a random order; each color was tested 10 times per mosquito, and the experiments lasted less than an hour (*n* = 30 mosquitoes).

For the electroantennogram (EAG) recordings, the female *An. stephensi* head was excised, the tip of each antenna was cut off with fine scissors, and the head was then mounted on the reference electrode composed of a 0.01” silver wire (A-M Systems, Carlsbord, WA, USA). The electrode was contained in a borosilicate-pulled capillary filled with a 1:3 mix of saline and electrode gel to protect the preparation from desiccation during the experiment. The head was mounted by the neck on the reference electrode. The preparation was then moved to the recording system with the tips of the antennae inserted into a recording electrode identical to the reference electrode. The mounted antennae were oriented at 90° from the main airline, which was carrying filtered air (Praxair, Danbury, CT, USA). The odor stimulus was delivered using a computer-controlled solenoid system, where filtered air was passed through a 2 mL odor cartridge containing the odorant. The odorant (2 μL of 1-octen-3-ol at 10^−4^ dilution in mineral oil, pipetted onto a filter paper) was delivered in 1 s pulses, 10 times per mosquito (*n* = 15 mosquitoes).

Odor and light-induced responses from the ERG and EAG recordings were amplified with an A-M Systems amplifier (10-100x; A-M Systems, 1800), digitized using a Digidata 1550B data acquisition system (Molecular Devices, San Jose, CA, USA) and analyzed using Matlab (MathWorks, Natick, MA, USA). EAG experiments used mosquitoes primed for 3 days with 10 nM histamine and control mosquitoes. For ERG experiments, we used histamine-primed mosquitoes (Section 2.3.1), and mosquitoes provisioned with histamine in an RBC-serum meal (Section 2.3.3).

#### 2.4.4. Histamine Immunohistochemistry

Tissues (head, thorax, and abdomen) were excised and fixed in 4% N-3-dimethylaminopropyl-N′-ethylcarbodiimide in 0.01 M phosphate-buffered saline (PBS) at 4 °C for 4 h. The tissue was then transferred and fixed in 4% paraformaldehyde in PBS overnight. Following fixation, tissues were washed in PBS and embedded in 7% agarose and sectioned at 100 μm using a Leica VT 1200S vibratome. Tissues were washed in PBS with 0.5% TritonX (PBT) and blocked in 2% goat serum for 1 h. Tissues were then incubated in 1:500 rabbit anti-histamine with 2% goat serum in PBT for 2 d at 4 °C. After primary antibody incubation, tissues were washed in PBT, blocked in goat serum, and incubated in 1:1000 Alexa Fluor 488. Tissues were then washed in PBT and PBS, processed through an ascending glycerol gradient and mounted in Vectashield^®^PLUS. The histamine antibody has shown to be specific for histamine and effective in a variety of invertebrate species, including *Drosophila melanogaster* [7,8,9]. Preabsorption controls with histamine eliminated any labeling.

### 2.5. Impact of Histamine Provisioning on Lifespan and Patterns of Blood Feeding of Uninfected Female An. stephensi over Time

A total of 120 female mosquitoes were placed into each of three cartons. Mosquitoes in these cartons were offered a histamine-treated or control RBC-serum meal once per week as described in Section 2.3.3. Unfed mosquitoes were removed after the first bloodmeal to ensure all mosquitoes at the beginning of the experiment imbibed the first treatment. Control and histamine-treated bloodmeals were offered once weekly to each group until no mosquitoes remained alive. At each blood-feeding, fed and unfed mosquitoes were counted and recorded. Mosquitoes were provided with an oviposition substrate at 3 d post-feeding, which was removed the following day. Dead females were counted and removed from each group four times per week. Two biological replicates were performed with separate cohorts of *An. stephensi*.

### 2.6. Impact of Histamine Provisioning on Plasmodium Infection of An. stephensi

#### 2.6.1. *P. y. yoelii* 17XNL Infection of *An. stephensi* and Blood Feeding Behavior of Infected Female Mosquitoes

Mosquitoes were primed for 3 d (Section 2.3.1) prior to feeding on infected mice as described in Section 2.3.4. Unfed mosquitoes were removed and discarded. To quantify infection prevalence and intensity, 25–35 mosquito midguts were dissected at 10 d PI and stained with mercurochrome to count midgut oocysts. For midgut infection analysis, we performed 10 biological replicates with separate cohorts of *An. stephensi*. Salivary glands were dissected from 12–18 mosquitoes per group at 12–15 d PI, with infections scored on a scale of 1–4 per pair of glands, with 1 for 100–1000 sporozoites, 2 for 1000–10,000 sporozoites, 3 for 10,000–100,000 sporozoites and 4 for 100,000+ sporozoites. Salivary gland infections were analyzed from 6 of the 10 replicate infections used for midgut oocyst analysis.

To examine the tendency to take a subsequent bloodmeal, two subsets of 15–20 blood-fed infected mosquitoes from each treatment group were placed in new, separate pint cartons. Mosquitoes were provided with an oviposition substrate at 3 d post-feeding, which was removed the following day, and then offered a second bloodmeal as described in Section 2.3.2 at 4 d or 11 d PI. The number of fed and unfed mosquitoes was counted and recorded. After this assay, mosquitoes were frozen and not used for other analyses. These studies were completed using mosquitoes from 5 of the 10 replicates prepared above for analyses of *P. y. yoelii* 17XNL midgut and salivary gland infection.

#### 2.6.2. Impact of Histamine Treatment on *P. falciparum* NF54 Growth in vitro

*Plasmodium falciparum* NF54 culture was initiated at 0.5% ring stages and maintained as described in Section 2.3.5. Treatments were prepared in duplicate, including 12 nM chloroquine as a positive control for parasite killing, along with 1, 10, and 100 nM histamine, all of which were changed daily. Plates were maintained in a candle jar [37] with samples collected from each well immediately after treatment (0 h) and 48 h and 96 h post treatment and stained with 12 μM Hoechst and 12 μM JC-1 to quantify parasitemia with flow cytometry.

#### 2.6.3. *P. falciparum* Infection of *An. stephensi*

*P. falciparum* NF54 gametocyte culture was initiated at 0.7% parasitemia if synchronized rings were present or at 0.5% parasitemia if mixed stages were present. Parasites were maintained as described in Section 2.3.5. Cultures at days 14, 15, and 17 and stage IV and V gametocytes were combined and used for infections [38]. Exflagellation was evaluated on the day of feeding before the addition of fresh media. Immediately before feeding, histamine was added to the infected bloodmeal as described in Section 2.3.3. Mosquitoes were fed according to Section 2.3.2, after which unfed mosquitoes were removed and discarded. At 10 d PI, midguts were dissected and stained with 0.5% mercurochrome to count midgut oocysts. Four biological replicates were performed with separate cohorts of *An. stephensi*. Salivary glands were dissected at 15 d PI, with infections scored on a scale of 1–4 per pair of glands, with 1 for 100–1000 sporozoites, 2 for 1000–10,000 sporozoites, 3 for 10,000–100,000 sporozoites and 4 for 100,000+ sporozoites. Salivary gland infections were analyzed from 11–18 mosquitoes per treatment group from one replicate used for midgut oocyst analysis.

### 2.7. Statistical Analyses

Data were analyzed using GraphPad (version 9.0.1, San Diego, CA, USA) or MatLab 2019a release. The numbers of mosquitoes taking a second bloodmeal and infection prevalence were analyzed using the Chi-square test. Infection intensity data were analyzed using the one-way Kruskal–Wallace ANOVA with post hoc Tukey. The EAG and ERG experiments were analyzed using an unpaired *t*-test or the Kruskal–Wallis test. For flight tunnel studies, mean mosquito flight velocities were calculated from the 3D tracks of each individual trajectory and data were analyzed using the Kruskal–Wallis or Mann–Whitney U-test with Bonferroni correction. Lifespan data from individual replicates were analyzed using the Kaplan–Meier analysis of survival with the log-rank test to compare groups. The proportions of mosquitoes feeding over time were analyzed using Kaplan–Meier analysis as the time to failure to feed; controls (no histamine) were not significantly different by the Wilcoxon test, so replicate data were combined for analysis. Cultured *P. falciparum* NF54 growth data were analyzed using ANOVA. *Plasmodium falciparum* salivary gland infection prevalence data were analyzed using the Chi-square test on proportional data, as several values were <5. For all analyses, significance was assumed at *p* ≤ 0.05.

## 3. Results

### 3.1. Histamine Provisioning of Uninfected An. stephensi Was Associated with Distinct Temporal Patterns in the Tendency to Take a Second Bloodmeal

**Figure 1:**
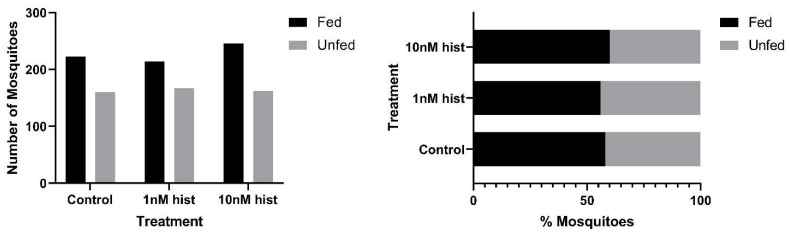
The tendency of histamine-primed uninfected *An. stephensi* to take a second bloodmeal 4 d later. **Left**: numbers of fed and unfed uninfected mosquitoes by treatment. **Right** panel: data from the left panel shown as percentages of fed and unfed uninfected mosquitoes in each group. There were no significant effects of treatment. N = 5 replicates; Chi-square test (α = 0.05); hist: histamine.

**Figure 2:**
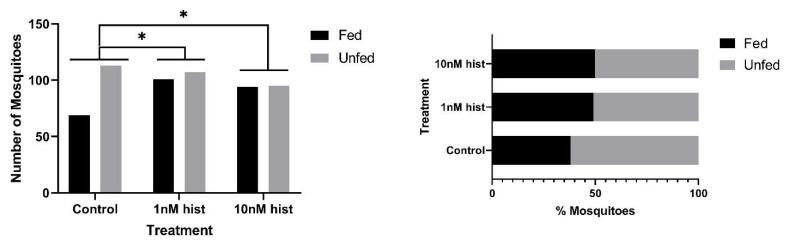
The tendency of histamine-primed, uninfected *An. stephensi* to take a second bloodmeal 14 d later. **Left**: numbers of fed and unfed uninfected mosquitoes by treatment with significant differences noted among pairs. **Right** panel: data from the left panel shown as percentages of fed and unfed uninfected mosquitoes in each group. N = 5; Chi-square test (α = 0.05); hist: histamine. * Control vs. 1 nM *p* = 0.034, control vs. 10 nM *p* = 0.022.

**Figure 3:**
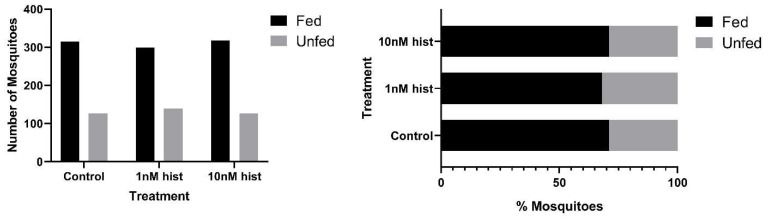
The tendency of uninfected *An. stephensi* provisioned with histamine (hist) in blood to take a second bloodmeal 4 d later. **Left**: numbers of fed and unfed uninfected mosquitoes by treatment. **Right** panel: data from the left panel shown as percentages of fed and unfed infected mosquitoes in each group. N = 5; Chi-square test (α = 0.05).

**Figure 4:**
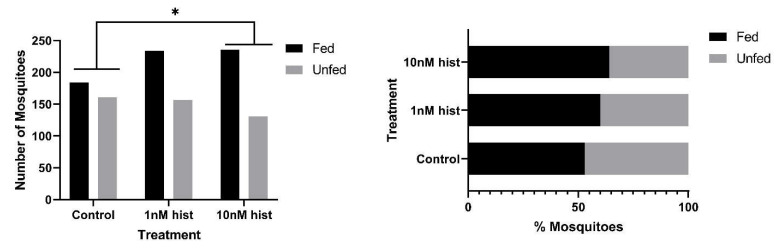
The tendency of uninfected *An. stephensi* provisioned with histamine (hist) in blood to take a second bloodmeal 14 d later. **Left**: numbers of fed and unfed uninfected mosquitoes by treatment with significant differences noted among pairs. **Right** panel: data from the left panel shown as percentages of fed and unfed uninfected mosquitoes in each group. N = 5; Chi-square test (α = 0.05). * *p* = 0.003.

In malaria-endemic areas, the proportions of uninfected mosquitoes in natural populations are high. In sub-Saharan Africa, where the endemicity of falciparum malaria is high and the primary vectors are anthropophilic, sporozoite infection rates are low, typically 10% and often less than 5% [39,40]. Further, infection of mosquitoes even after direct skin feeding on infected falciparum gametocyte-positive volunteers is far from uniform; the average success rate of *Anopheles gambiae* and *Anopheles arabiensis* infection from a survey of 930 feeding experiments in a variety of endemic settings was 62% [41]. Accordingly, some proportion of vector mosquitoes consumes blood from infected individuals without becoming infected. To examine the effects of provisioning normal blood histamine (1 nM) and severe malaria-associated blood histamine (10 nM) on uninfected *An. stephensi*, we first examined the tendency to take a second bloodmeal at 4 d or 14 d later, behavior that would impact the likelihood of becoming infected at a subsequent bloodmeal. These timepoints reflect the mid-point and completion of the extrinsic incubation period, respectively, for *Plasmodium* spp. sporogony. Histamine priming had no effect on the tendency to take a second bloodmeal 4 d after the first bloodmeal (Figure 1). However, priming with both 1 nM and 10 nM histamine was associated with increased tendencies to take a second bloodmeal at 14 d after the first bloodmeal relative to control uninfected *An. stephensi* (Figure 2). The effects of blood delivery of histamine were similar to those of histamine priming. Specifically, blood-delivered histamine had no effect on the tendency to take a second bloodmeal 4 d later (Figure 3), but there was an increased tendency to take a second bloodmeal at 14 d later in mosquitoes provisioned with 10 nM histamine relative to control (Figure 4). To summarize, uninfected mosquitoes responded to both doses of histamine delivered by priming with an increased tendency to take a second bloodmeal at 14 d (Figure 2), while the delivery of histamine in the blood increased this tendency at 14 d at the treatment dose associated with severe malaria (10 nM; Figure 4).

### 3.2. Histamine Provisioning Enhanced Uninfected Female An. stephensi Flight Activity and Physiological Responses to Olfactory Cues

**Figure 5:**
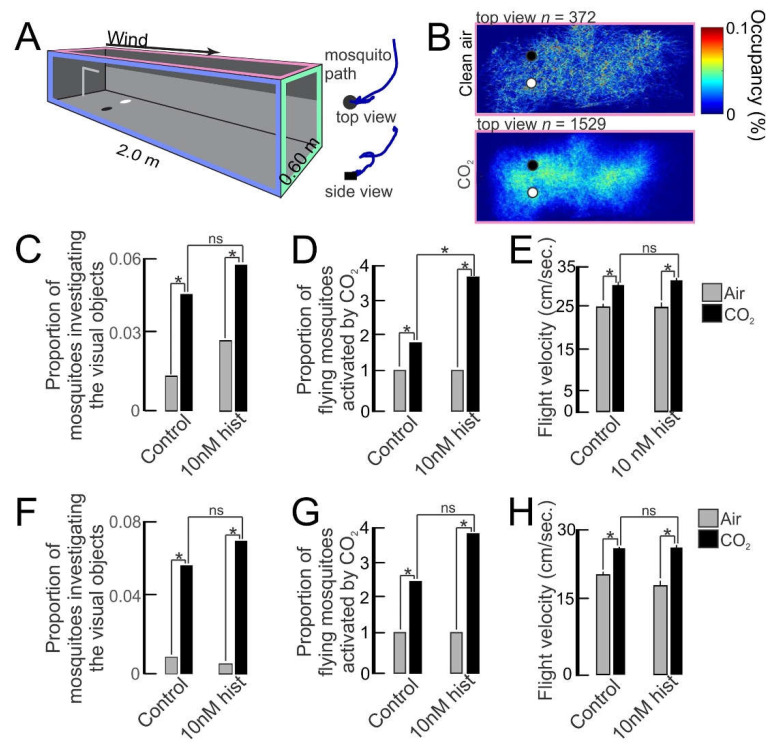
The flight activity of *An. stephensi* and the response to visual objects with histamine (hist) priming or provisioning in a bloodmeal. (**A**) Wind tunnel with real-time tracking to quantify mosquito behavior to olfactory and visual cues. (Right) Top and side views of a mosquito trajectory investigating the visual object (black circle). (**B**) Occupancy maps of mosquito activity during filtered air (top) and CO_2_ exposure (bottom). (**C**–**E**) Investigation of visual objects (**C**), flight activity (**D**), and flight velocities (**E**) of histamine-primed and control mosquitoes to filtered air and CO_2_ treatments. * *p* < 0.05 (Wilcoxon rank test for **C** and **D**, *t*-test for **E**); (ns) denotes not significantly different (*p* > 0.05). (**F**–**H**) Mosquitoes provisioned with 10 nM histamine in a bloodmeal or control mosquitoes (no histamine in the bloodmeal) and their investigation of visual objects (**F**), flight activity (**G**), and flight velocities (**H**). * *p* < 0.05 (Wilcoxon rank test for **F** and **G**; *t*-test for **H**); (ns) denotes not significantly different (*p* > 0.05).

The tendency to take a second bloodmeal is likely to be linked to responses to visual and olfactory cues from the host. To test this directly, we primed female *An. stephensi* with 10 nM histamine (severe malaria) or diluent (water) in soaked cotton balls for 3 d; sugar cubes were provided as a nutrient source. On the day following priming, we flew the mosquitoes in a wind tunnel (Figure 5A) that allowed control of the airflow conditions (olfactory stimuli) and visual stimuli that mosquitoes might experience in nature. The occupancy map showed that during clean air treatment, few mosquitoes flew in the tunnel (Figure 5B, *n* = 372 for this replicate, top panel; *n* = 1944 total for all replicates). By contrast, CO_2_ exposure was associated with greater numbers of mosquitoes flying in the tunnel (Figure 5B, *n* = 1529 for this replicate, bottom panel; *n* = 6182 total for all replicates). In control and primed groups, the proportion investigating visual objects increased significantly from 1%–2% in clean air to 5%–6% during CO_2_ exposure (gray vs black bars, Figure 5C), but priming did not increase this behavior in CO_2_-exposed mosquitoes (control black bar vs histamine-primed black bar, Figure 5C). Priming, however, significantly increased CO_2_-induced flight activity compared to controls (control black bar vs histamine-primed black bar, Figure 5D). CO_2_ significantly increased the flight velocities of both control and 10 nM histamine-primed mosquitoes relative to clean air (gray vs black bars, Figure 5E), but priming did not increase this behavior in CO_2_-exposed mosquitoes (control black bar vs histamine-primed black bar, Figure 5E). In all studies, the behavior returned to the baseline post-CO_2_, *p* > 0.05.

We conducted similar experiments using *An. stephensi* provisioned with 10 nM histamine in an RBC-serum meal or an RBC-serum meal supplemented with an equivalent volume of water used as histamine diluent. For both control and histamine-fed mosquitoes, CO_2_ increased the number of mosquitoes investigating the visual objects (gray vs black bars, Figure 5F), but histamine treatment did not increase this behavior in CO_2_-exposed mosquitoes (control black bar vs histamine-fed black bar, Figure 5F). The CO_2_-induced flight activity relative to clean air was significantly increased in both control and histamine-treated mosquitoes (gray vs black bars, Figure 5G), but histamine treatment did not increase this behavior in CO_2_-exposed mosquitoes (control black bar vs histamine-fed black bar, Figure 5G). Like histamine-primed mosquitoes, the flight velocity of CO_2_-exposed mosquitoes was not increased by histamine provisioning in an RBC-serum meal relative to control mosquitoes (control black bar vs histamine-fed black bar, Figure 5H). Overall, histamine priming and provisioning did not increase the investigation of visual objects (Figure 5C,F) or flight velocities of CO_2_-exposed mosquitoes (Figure 5E,H), but flight activities of CO_2_-exposed mosquitoes relative to controls were significantly increased in primed mosquitoes (Figure 5D) and trended toward an increase in provisioned mosquitoes (Figure 5G).

**Figure 6:**
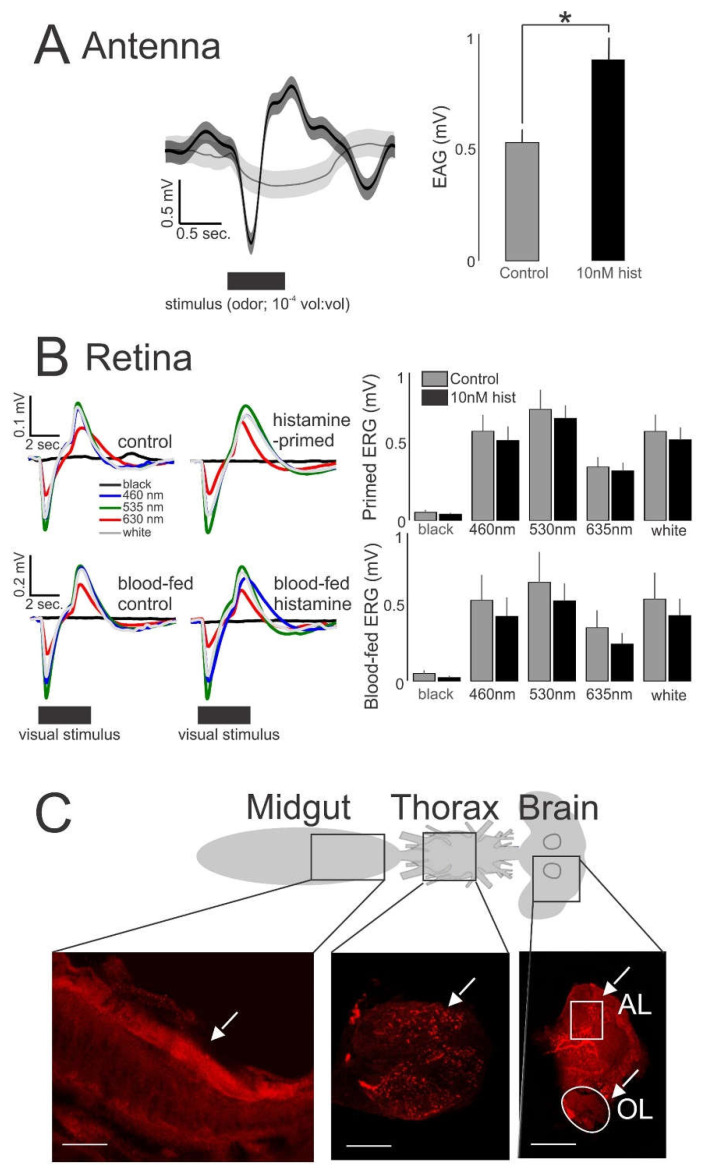
Electrophysiological recordings and histaminergic innervation of *An. stephensi* tissues. (**A**) Histamine (hist) priming caused increased EAG responses (*n* = 15) that were significantly greater than control (*t*-test: * *p* = 0.04). The traces show the mean response for the two treatment groups (left); the bars are means ± SE (right). (**B**) ERG responses (*n* = 30) to different light stimuli. Responses were not significantly different between histamine-treated (primed and blood-fed) and control groups (*t*-test: *p* > 0.05). Traces are responses to the light stimuli for the primed mosquitoes (left, top) and blood-fed mosquitoes (left, bottom). Bars are the means ± SE (right). (**C**) Schematic of *An. stephensi* tissues (midgut, thoracic ganglion, brain) that were immunolabeled for histamine. The white arrows denote the strong histamine staining (red) of the midgut lining (left, bottom) and neuropil in the thoracic ganglion (middle, bottom). Section of the brain shows strong immunolabeling of histamine in cell clusters immediately anterior to the antennal lobe (AL) and cells adjacent to the olfactory lobe (OL). White lines denote 100 μm distance.

To determine if behavioral responses were associated with changes in sensory physiology, we electrophysiologically recorded from the antenna and eye of a randomly selected sample of mosquitoes from behavioral experiments in Figure 5. We completed electroantennogram (EAG) and low light (70 μW/cm^2^/nm) electroretinogram (ERG) recordings, outputs that are thought to reflect the sum of receptor neuron responses of the antenna and eye, respectively. Histamine-primed mosquitoes exhibited significantly greater EAG responses to odor stimulation than control mosquitoes (Figure 6A). While histamine-primed, histamine blood-fed, and control mosquitoes exhibited significant ERG responses to the tested wavelengths (Kruskal–Wallis test, *p* < 0.01), with the greatest response to green (530 nm), followed by white, blue (460 nm), and red (635 nm) relative to a black no-stimulus control (Figure 6B), histamine delivery via priming or RBC-serum meal did not increase these responses relative to control. To place these sensory physiological responses in a tissue context consistent with ingestion of histamine, we confirmed histamine staining (Figure 6C) in the midgut, thoracic ganglion, and visual and olfactory regions of the brain. The midgut showed strong staining in the interior (Figure 6C, arrow, left panel). The thoracic ganglion showed bleb-like tracts throughout the ganglion (Figure 6C, arrow, middle panel), while the brain showed strong staining in loci associated with the antennal lobe (AL) and optic lobe (OL) (Figure 6C, arrows, right panel). Taken together, our behavioral and electrophysiological assays suggested an association between increased olfactory responsiveness (Figure 6A) and increased flight activity in CO_2_-exposed, histamine-primed mosquitoes (Figure 5D). Histamine treatment, however, had no effect in CO_2_-exposed mosquitoes on the response to visual cues relative to controls (Figure 5C,F), and this correlated with a lack of histamine-enhanced ERG responses (Figure 6B).

### 3.3. Histamine Provisioning Had Modest to No Effects on Uninfected Female An. stephensi Lifespan, But Increased the Tendency to Take Weekly Bloodmeals with Increasing Age

**Figure 7:**
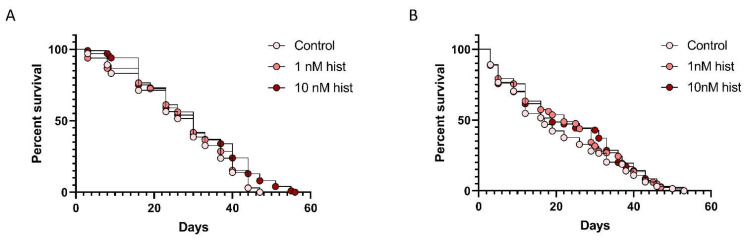
Percent survival of uninfected mosquitoes provisioned with 1 nM histamine (hist) and 10 nM histamine or with an equivalent volume of water diluent (control) in a weekly bloodmeal. (**A**) Replicate 1. The survival of mosquitoes provisioned with 10 nM histamine was significantly different from the survival of control mosquitoes and mosquitoes provisioned with 1 nM histamine. Kaplan–Meier with log-rank test (α = 0.05); control vs 10 nM histamine, *p* = 0.0242. (**B**) Replicate 2. There were no significant differences in survival among treatments. Kaplan–Meier with log-rank test (α = 0.05).

**Figure 8:**
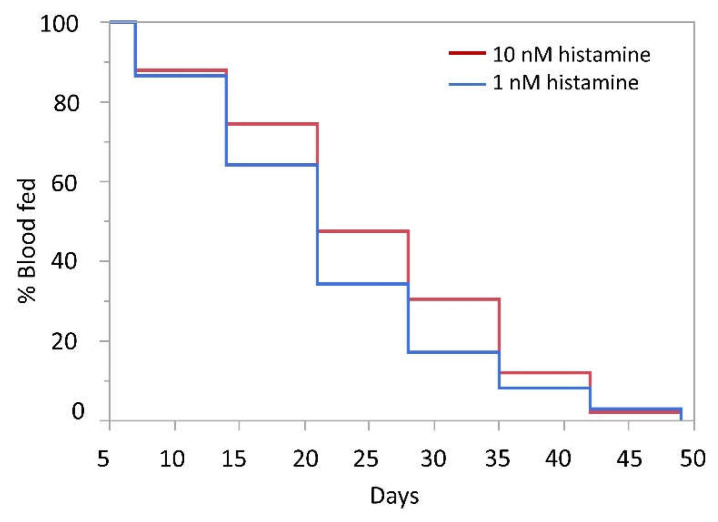
Percent feeding of uninfected mosquitoes provisioned with 1 nM histamine or 10 nM histamine in weekly bloodmeals over time. A greater proportion of mosquitoes that were provisioned with 10 nM histamine fed on the RBC-serum meal compared with mosquitoes that were provisioned with 1 nM histamine each week. N = 2; Wilcoxon test (α = 0.05), 1 nM vs 10 nM histamine, *p* = 0.0189.

A recent study of *An. stephensi* suggested that mosquito survival to 35–60 days occurs across a broad temperature range [42]. The authors also showed that the proportion of females blood-feeding declined with increasing mosquito age, so an increase in feeding tendency at a more advanced age promoted by ingested histamine could partially offset age-related declines in blood-feeding to promote parasite infection and transmission. Based on these observations and the fact that mosquito longevity is perhaps the most important variable for vectorial capacity, we examined the survival of female *An. stephensi* that were offered weekly RBC-serum meals supplemented with 1 nM or 10 nM histamine or RBC-serum meals supplemented with a volume of water equivalent to that used for the addition of histamine. In one replicate, mosquitoes that received weekly meals supplemented with 10 nM histamine lived significantly longer than controls (Figure 7A), with increased survival apparent beyond 42 d. In a second replicate (Figure 7B), no differences in survival were detected, affirming that histamine effects on *An. stephensi* lifespan are modest to negligible. Given that blood-feeding tendency with increasing age directly impacts parasite transmission, we also examined the proportion of mosquitoes in each group that blood-fed each week (Figure 8). The proportional feeding data from these replicates were combined for analysis because the Kaplan–Meier test showed no difference (*p* = 0.0631) between mean times to failure to feed for the control in replicate 1 (23.04 days) and the control in replicate 2 (26.62 days). From weeks 2–6, a greater proportion of mosquitoes that were provisioned with 10 nM histamine fed on the RBC-serum meal compared with mosquitoes that were provisioned with 1 nM histamine each week (Figure 8). These results indicate that weekly exposure to 10 nM histamine can increase the tendency of *An. stephensi* to bloodfeed even at extended to ages in a manner that is not dependent on changes in survivorship.

### 3.4. Histamine Provisioning Enhanced An. stephensi Infection with P. y. yoelii 17XNL

**Figure 9:**
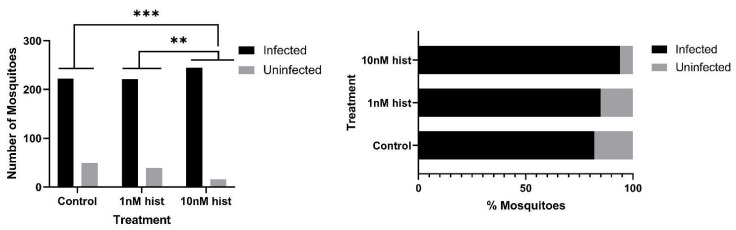
Proportions of *An. stephensi* infected with *P. y. yoelii* 17XNL oocysts following priming for 3 d prior to infection with histamine (hist) in water or water only via soaked cotton balls. **Left**: numbers of infected and uninfected mosquitoes by treatment with significant differences noted among pairs. **Right** panel: data from the left panel shown as percentages of uninfected and infected mosquitoes in each group. N = 10; Chi-square test (α = 0.05). *** *p* = 4.6 × 10^−5^, ** *p* = 0.0016.

**Figure 10:**
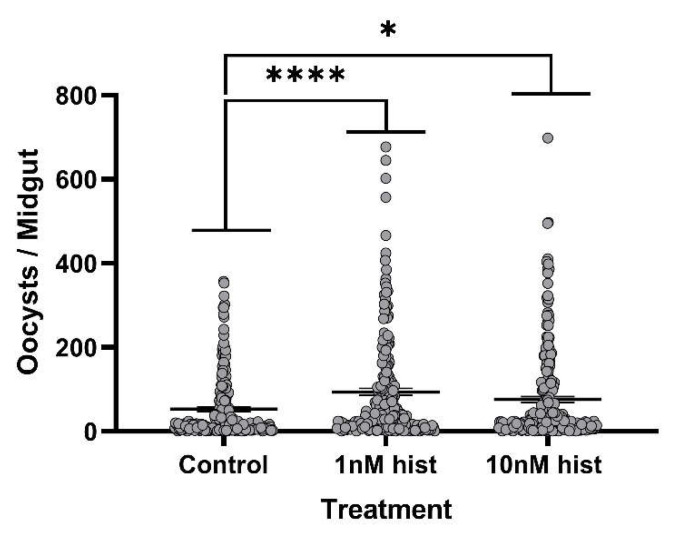
The mean *P. y. yoelii* 17XNL midgut oocysts in *An. stephensi* following priming for 3 d prior to infection with histamine (hist) or water (control) via soaked cotton pads. N = 10; one-way ANOVA (α = 0.05). **** *p*-value = 0.00012, * *p* = 0.042.

**Figure 11:**
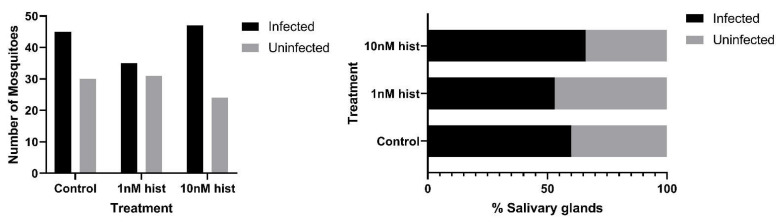
Proportions of *An. stephensi* with *P. y. yoelii* 17XNL salivary gland sporozoites following priming for 3 d prior to infection with histamine (hist) or water (control) via soaked cotton pads. **Left**: numbers of mosquitoes with infected and uninfected salivary glands. **Right** panel: data from the left panel shown as percentages of uninfected and infected mosquitoes in each group. N = 6; Chi-square test (α = 0.05), no significance.

**Figure 12:**
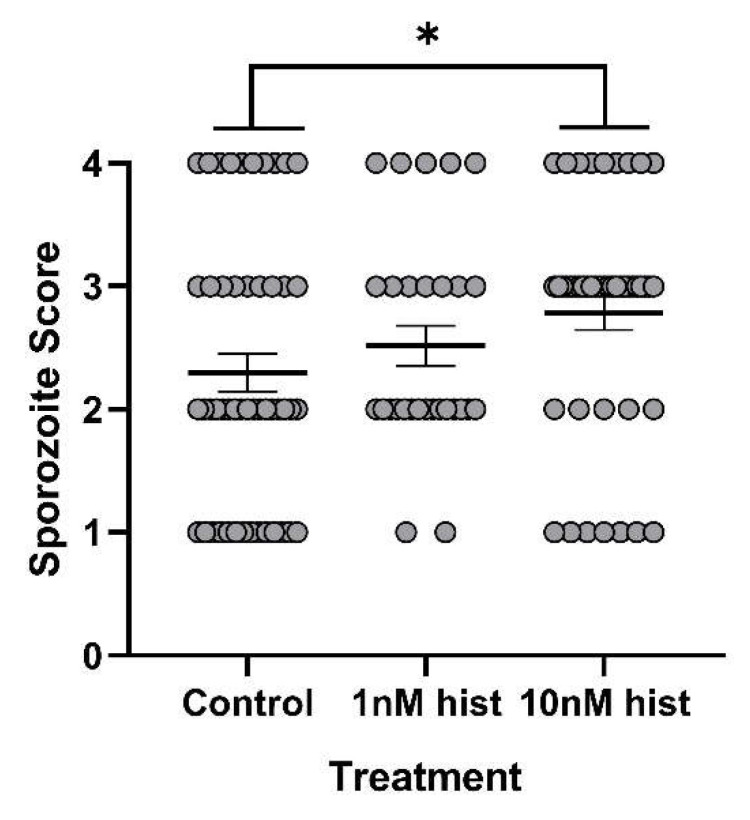
The mean salivary gland score in *An. stephensi* infected with *P. y. yoelii* 17XNL sporozoites following priming for 3 d prior to infection with histamine (hist) or water (control) via soaked cotton pads. N = 6; one-way ANOVA (α = 0.05). * *p*-value = 0.00484.

The tendency for a mosquito to become infected is obviously critical for transmission, but given the fact that some studies have suggested that sporozoite density and infectivity decline over the first four weeks of salivary gland infection [43], the intensity of infection should not be overlooked in considering the likelihood of transmission with increasing mosquito age. For these studies, we used CD1 outbred mice, which have low basal plasma histamine levels (20–30 nM [44]) and do not exhibit increased circulating histamine in the peripheral blood in response to *P. y. yoelii* 17XNL infection (Figure S1). With histamine priming, the proportions of mosquitoes with at least one oocyst per midgut were significantly higher in the 10 nM histamine group compared to both control and 1 nM histamine-treated mosquitoes (Figure 9). Further, histamine priming was associated with significantly increased mean oocysts per infected midgut compared to controls (Figure 10). Histamine priming had no effect on the proportions of mosquitoes with infected salivary glands (Figure 11), but salivary gland sporozoite density was significantly increased with 10nM histamine priming relative to controls (Figure 12).

### 3.5. Histamine Provisioning Had No Effect on the Tendency of P. y. yoelii 17XNL-Infected An. stephensi to Take a Subsequent Bloodmeal

**Figure 13:**
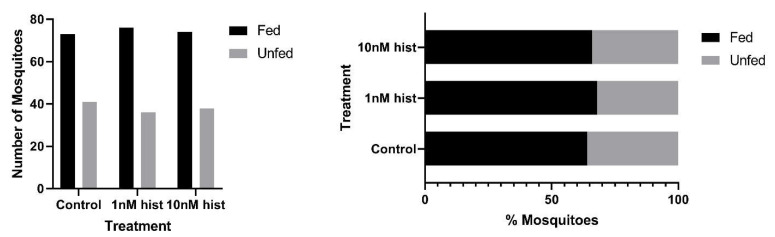
Tendency of histamine-primed *An. stephensi* infected with *P. y. yoelii* 17XNL to take a second bloodmeal at 4 d post-infection (PI). **Left**: numbers of fed and unfed infected mosquitoes by treatment. **Right** panel: data from the left panel shown as percentages of fed and unfed infected mosquitoes in each group. N = 5; Chi-square test (α = 0.05); hist: histamine.

**Figure 14:**
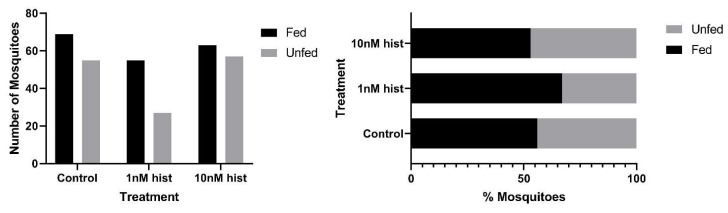
Tendency of histamine-primed *A. stephensi* infected with *P. y. yoelii* 17XNL to take a second bloodmeal at 11 d post-infection (PI). **Left**: numbers of fed and unfed infected mosquitoes by treatment. **Right** panel: data from the left panel shown as percentages of fed and unfed infected mosquitoes in each group. N = 5; Chi-square test (α = 0.05); hist: histamine.

The tendency to take a second bloodmeal by an infected mosquito is of obvious importance for transmission. However, the timing of the second meal is critical. If the meal is taken during oocyst infection, there is a risk that the mosquito could die in the bout with no resulting parasite transmission. Accordingly, we selected two time points for these studies: 4 d PI when only oocysts were present or 11 d PI when sporozoites were present in the glands. Histamine priming had no effect on the tendency to take a second bloodmeal by infected *An. stephensi* at either 4 d or 11 d PI (Figure 13 and Figure 14). Combined with the data above, ingestion of elevated levels of histamine (10 nM) associated with severe malaria increased the tendency of uninfected *An. stephensi* to take a second bloodmeal and increased midgut and salivary gland infection success with *P. y. yoelii* 17XNL, but in the presence of parasite infection, histamine provisioning did not increase the tendency to take a second bloodmeal.

### 3.6. Histamine Provisioning Enhanced An. stephensi Infection with P. falciparum NF54 Strain

**Figure 15:**
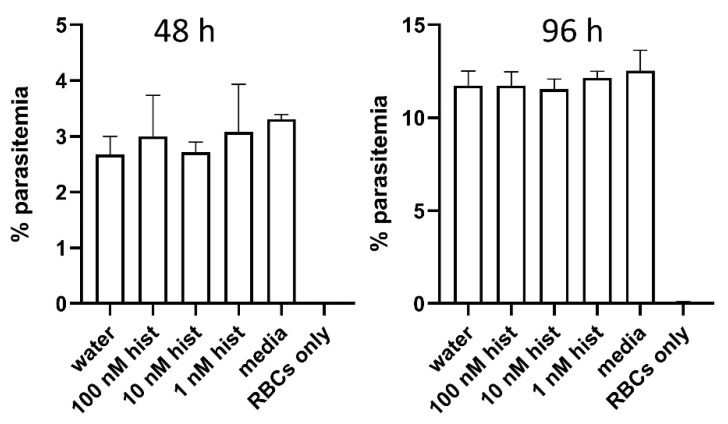
Histamine treatment of *P. falciparum* NF54 in vitro had no significant direct effects on parasite growth. **Left**: percent of parasitemias at 48 h (one growth cycle) following treatment with 1, 10, and 100 nM histamine (hist), an equivalent volume of water used to deliver histamine treatments or only media added to parasites. Human RBCs were included to indicate the specificity of flow cytometric detection of fluorescence. **Right** panel: parasites from the left panel at 96 h after treatment. N = 4 replicates analyzed per treatment, and each analyzed in duplicate; ANOVA (α = 0.05).

**Figure 16:**
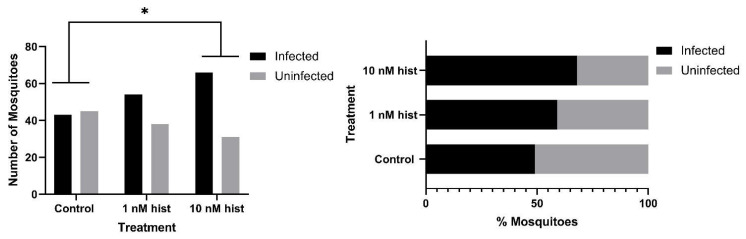
Proportions of *An. stephensi* infected with *P. falciparum* NF54 oocysts with histamine (hist) provisioning in the infected bloodmeal. **Left**: numbers of infected and uninfected mosquitoes by treatment with significant differences noted among pairs. **Right** panel: data from the left panel shown as percentages of uninfected and infected mosquitoes in each group. N = 4; Chi-square test (α = 0.05). * *p* = 0.0108.

**Figure 17:**
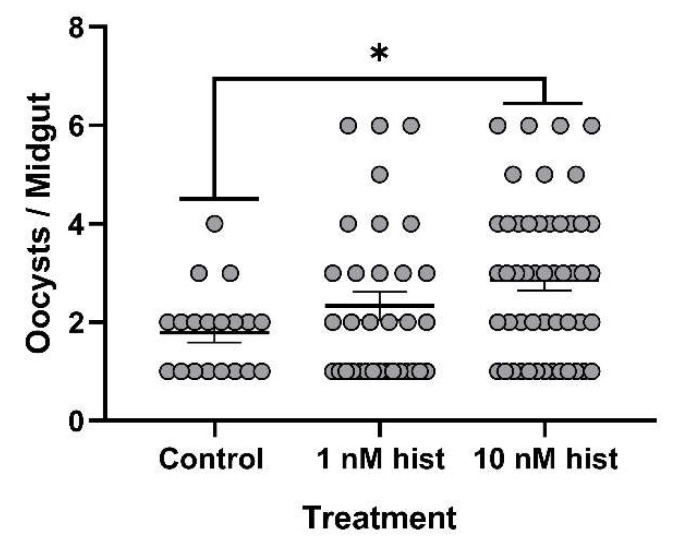
Mean *P. falciparum* NF54 midgut oocysts in *An. stephensi* with histamine (hist) provisioning in the infected bloodmeal. N = 4; one-way ANOVA (α = 0.05). * *p*-value = 0.0152.

**Figure 18:**
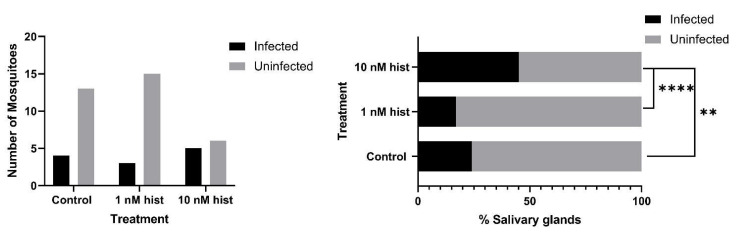
Proportions of *An. stephensi* with *P. falciparum* NF54 salivary gland sporozoites with histamine (hist) provisioning in the infected bloodmeal. **Left**: numbers of mosquitoes with infected and uninfected salivary glands. **Right** panel: data from the left panel shown as percentages of uninfected and infected mosquitoes in each group. N = 1; Chi-square test (α = 0.05). ** *p* = 0.0028, **** *p* < 0.0001.

To establish whether observations with a mouse malaria parasite could be extended to infection with a human parasite, we examined the infection success of *An. stephensi* using cultured *P. falciparum* NF54. In vitro culture of this parasite also allowed us to pose an important question: could malaria parasites respond directly to histamine, suggesting that histamine might concurrently alter both mosquito and parasite biology during infection of the mosquito host? To address this question, we treated parasites in a culture based on our established drug assay protocol [32,45,46,47,48], which we modified for the use of Hoechst and JC-1 stains versus propidium iodide. Based on the results of these assays, we confirmed that *P. falciparum* growth is not directly altered by histamine at the concentrations used in our assays (Figure 15). While asexual parasites in culture are not biologically equivalent to mosquito-stage parasites, the lack of encoded receptors for histamine in the genomes of *Plasmodium* spp. supports our assumption that histamine treatment of infected *An. stephensi* alters mosquito biology only. For parasite infection studies, female *An. stephensi* were allowed to feed on gametocyte culture, to which histamine was added to final concentrations of 1 nM or 10 nM immediately before blood-feeding. An aliquot of the same parasite culture was supplemented as a control with a volume of water equivalent to that used to add the histamine treatments. With histamine provisioning, the proportion of mosquitoes with at least one oocyst per midgut was significantly increased in the 10 nM histamine group compared to control mosquitoes (Figure 16). Priming with 10 nM histamine was also associated with increased mean oocysts per infected midgut compared to control mosquitoes (Figure 17). The proportion of mosquitoes with salivary gland sporozoites was significantly higher with provisioning of 10 nM histamine compared to both control and 1 nM histamine treated mosquitoes (Figure 18), but there was no effect on sporozoite density, as all were a score of 1. Together with the observations above, these data suggest that elevated histamine (10 nM) associated with severe malaria promotes infection of a single mosquito host with both *P. y. yoelii* 17XNL and *P. falciparum* parasites with notably divergent genetics and life cycle biology [49].

## 4. Discussion

**Figure 19:**
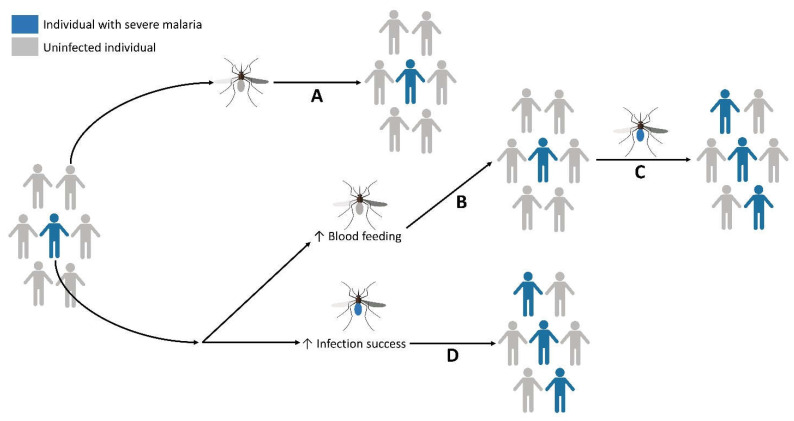
Ingestion of histamine at levels associated with severe malaria can enhance *An. stephensi* behaviors and parasite infection success in a manner that would be expected to amplify parasite transmission to and from human hosts. (**A**) If a female mosquito takes a bloodmeal from an uninfected individual (gray host), then subsequently feeds on individuals in the same population, malaria incidence is not increased. (**B**) If a female mosquito takes a bloodmeal from an individual with severe malaria (blue host), that mosquito may not become infected even if the individual is gametocytemic. However, ingestion of elevated blood histamine (10 nM) by this uninfected mosquito would be associated with increased flight activity, responsiveness to host cues and tendency to take a second bloodmeal from this host population, providing an increased opportunity for this mosquito to become infected (**C**). Importantly, our observations of increased feeding tendency at a more advanced age promoted by ingested histamine could also partially offset age-related declines in blood-feeding to promote parasite infection and transmission. Once infected, this mosquito can transmit parasites to new hosts, increasing the incidence of malaria in the population. Mosquitoes that become infected after feeding on a gametocytemic host with severe malaria and elevated blood histamine (**D**) are more likely to transmit parasites to humans because an increased proportion carries salivary gland sporozoites. Figure created with BioRender.com.

Together, our data show that histamine ingestion alters the physiology and behavior of uninfected and *Plasmodium-*infected *An. stephensi* in a manner that would be expected to amplify malaria parasite transmission (Figure 19). To support this conclusion, we examined mosquito tendency to take a second bloodmeal, flight activity and investigation of visual objects, neurophysiological responses to odor and light stimuli, lifespan and blood-feeding with increasing age, and infection success with two biologically distinct parasite species. The use of two different concentrations of histamine, including a normal human blood level (1 nM) and a blood level associated with SFM (10 nM) delivered by priming in soaked cotton balls or provisioned in an RBC-serum meal, provided an overview of responses that likely result from histamine detection by *An. stephensi* midgut receptors, followed by signaling that is transduced via the peripheral and central nervous systems.

Both histamine priming and provisioning in blood increased flight activity of CO_2_-exposed uninfected *An. stephensi* and histamine priming increased neurophysiological responsiveness to host odors. Host cues, such as odor and visual cues, are sensed by mosquitoes at different distances from the host. CO_2_ may be detected by mosquitoes at 15–50 m away, whereas visual detection of hosts occurs at much closer distances, at approximately 1–5 m [34]. Histamine is an important neuromodulator and has been shown to selectively impact sensory processing in insects. For example, in certain crepuscular insects, such as *Manduca sexta*, there is histaminergic coupling between the flight motor system in the thoracic ganglion and the olfactory system in the brain. By contrast, in diurnal insects, like *Pieris rapae* and *D. melanogaster*, this coupling occurs between the thoracic ganglion and the visual system [9]. For crepuscular insects that rely on olfactory more than visual cues, this is thought to increase their sensitivity to important odors. Intriguingly, in this study, we found that histamine treatment in *An. stephensi* increased the proportion of mosquitoes activated by CO_2_ but did not increase their attraction to visual objects in the wind tunnel at 4 d after treatment. We also found that histamine treatment did not affect retinal (ERG) responses to specific wavelengths and white light. In concert with these findings, we found strong histaminergic staining in the midgut and certain areas of the brain, including strong innervation near the antennal and olfactory lobes. These observations may explain why histamine-treated, uninfected mosquitoes did not exhibit an increased tendency to take a second bloodmeal at 4 d after the first. Additional studies are underway at later time points (i.e., 11 d and 14 d after the first bloodmeal) to fully understand the connections between neurophysiological responsiveness to histamine and these complex behaviors.

Weekly provisioning of 10 nM histamine significantly increased the tendency to blood feed from week 2 to week 6 of lifespan relative to 1 nM histamine treated mosquitoes. Longevity and human biting rate have the greatest impacts on vectorial capacity [50], so this persistent response to histamine that is independent of effects over the same period of time on survivorship is striking and likely to amplify parasite infection and transmission by *An. stephensi*. These observations provide further support for our inferences that ingested histamine could offset age-related declines in blood-feeding by directly increasing the tendency to blood feed at advanced ages.

Priming with 10 nM histamine increased the proportion of *An. stephensi* females infected with *P. y. yoelii* 17XNL, increased mean midgut oocysts, and increased sporozoite density in the salivary glands relative to controls. Similarly, provisioning of 10 nM histamine in a *P. falciparum*-infected bloodmeal significantly increased the proportion of mosquitoes with midgut oocysts, increased mean oocysts per midgut, and increased the proportion of females with salivary gland sporozoites. Given that additional blood-feeding has been observed to increase *P. falciparum* oocyst size and shorten the time to sporozoite appearance in the salivary glands of *Anopheles gambiae*, changes that could decrease the parasite extrinsic incubation period [51,52], we examined the tendency of *P. y. yoelii* 17XNL-infected mosquitoes to take a second bloodmeal. Our data suggested that histamine had no effect on the tendency of infected mosquitoes to take a second bloodmeal at 4 d or 11 d PI. Ingestion of histamine during a feeding bout by an uninfected mosquito, however, on an individual with SFM that did not result in mosquito infection would be predicted to increase the tendency to take a subsequent bloodmeal, and the chances of becoming infected later in adult life. In addition to these factors and in light of the potential for declining levels of salivary gland sporozoites after four weeks [43], increased prevalence and intensity of infection with oocysts and sporozoites could further enhance transmission by older mosquitoes.

While we have focused our studies on elevated histamine levels associated with SFM, parasites that are known to co-infect individuals with malaria can also increase circulating blood histamine and increase malarial gametocytemia [53,54,55,56,57]. In this context, anopheline mosquitoes feeding on co-infected patients could exhibit similar changes in physiology and parasite infection success that we have reported here. Co-infections with malaria are clinically, epidemiologically and physiologically complex with outcomes and transmission of the co-infecting malaria parasites driven by the timing of co-infection, host immunity, and the species of co-infecting parasites [53,54,55,56,57]. In this context, it is interesting to speculate that circulating blood histamine levels could function as a predictive biomarker for the risk of malaria parasite transmission in complex clinical co-infections with malaria.

In the mammalian host, malaria can trigger a T helper 2 (Th2)-type immune response characterized by elevated blood levels of histamine [2,28,58,59,60,61]. In general, a balance between both Th1- and Th2-type immune responses is required for optimal clearance of parasites and to prevent excessive immunopathology due to an overwhelming Th1 response [24,58,61,62]. Importantly, a Th2-biased response has been associated with reduced gametocyte killing and has been shown in animal models to increase parasite transmission to mosquitoes [63,64,65]. The malaria parasite benefits, therefore, from host manipulation that increases circulating histamine through enhanced survival in the mammalian host and enhanced transmission to mosquitoes. We would suggest that our data demonstrate that enhanced transmission would be extended by direct effects of ingested histamine on mosquito physiology and behavior. Such knowledge could be used to connect clinical interventions (e.g., reducing elevated histamine to mitigate human disease [28]) with the delivery of novel lures [66] to manipulate histamine signaling in vector mosquitoes for improved future malaria control. Accordingly, continued studies of the role of histamine in vector biology will provide novel insights into the complexity of the malaria parasite life cycle.

## Data Availability

“Wind tunnel data and code are available for download (Riffell, 2021).” Riffell, J.A. Code and data for wind tunnel and electrophysiology recordings. Available online: https://github.com/riffelllab (accessed on 10 May 2021).

## Supplementary Materials

The following are available online at https://www.mdpi.com/article/10.3390/biom11050719/s1, Figure S1. Plasma histamine in *P. y. yoelii* 17XNL-infected mice over the course of infection was not increased relative to control, uninfected mice.
