# Supplementary figures and images for: Histamine Ingestion by Anopheles stephensi Alters Important Vector Transmission Behaviors and Infection Success with Diverse Plasmodium Species
Source: Biomolecules. 2021 May 11;11(5):719. doi: 10.3390/biom11050719 (PMC8151525; doi:10.3390/biom11050719)

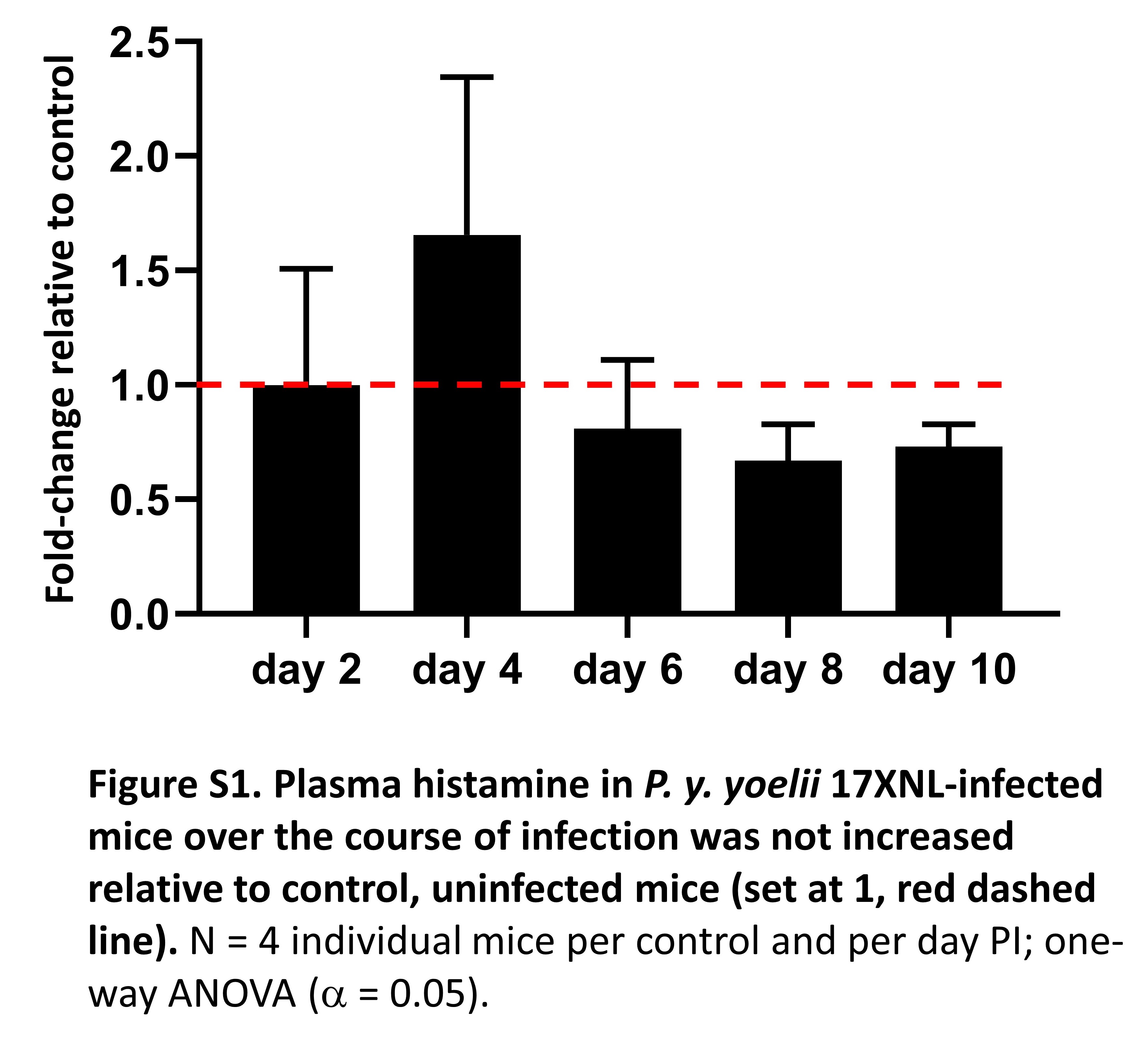

Supplement: Supplementary file 1 [file biomolecules-11-00719-s001.zip › biomolecules-1190464-supplementary.jpg]
